# The barley EST DNA Replication and Repair Database (bEST-DRRD) as a tool for the identification of the genes involved in DNA replication and repair

**DOI:** 10.1186/1471-2229-12-88

**Published:** 2012-06-14

**Authors:** Damian Gruszka, Marek Marzec, Iwona Szarejko

**Affiliations:** 1 Department of Genetics, Faculty of Biology and Environment Protection, University of Silesia, Jagiellonska 28, 40-032, Katowice, Poland

**Keywords:** Arabidopsis, Barley, Database, DNA replication, DNA repair, EST, Gene identification

## Abstract

**Background:**

The high level of conservation of genes that regulate DNA replication and repair indicates that they may serve as a source of information on the origin and evolution of the species and makes them a reliable system for the identification of cross-species homologs. Studies that had been conducted to date shed light on the processes of DNA replication and repair in bacteria, yeast and mammals. However, there is still much to be learned about the process of DNA damage repair in plants.

**Description:**

These studies, which were conducted mainly using bioinformatics tools, enabled the list of genes that participate in various pathways of DNA repair in *Arabidopsis thaliana* (L.) Heynh to be outlined; however, information regarding these mechanisms in crop plants is still very limited. A similar, functional approach is particularly difficult for a species whose complete genomic sequences are still unavailable. One of the solutions is to apply ESTs (Expressed Sequence Tags) as the basis for gene identification. For the construction of the barley EST DNA Replication and Repair Database (bEST-DRRD), presented here, the Arabidopsis nucleotide and protein sequences involved in DNA replication and repair were used to browse for and retrieve the deposited sequences, derived from four barley (*Hordeum vulgare* L.) sequence databases, including the “Barley Genome version 0.05” database (encompassing ca. 90% of barley coding sequences) and from two databases covering the complete genomes of two monocot models: *Oryza sativa* L. and *Brachypodium distachyon* L. in order to identify homologous genes. Sequences of the categorised Arabidopsis queries are used for browsing the repositories, which are located on the ViroBLAST platform. The bEST-DRRD is currently used in our project during the identification and validation of the barley genes involved in DNA repair.

**Conclusions:**

The presented database provides information about the Arabidopsis genes involved in DNA replication and repair, their expression patterns and models of protein interactions. It was designed and established to provide an open-access tool for the identification of monocot homologs of known Arabidopsis genes that are responsible for DNA-related processes. The barley genes identified in the project are currently being analysed to validate their function.

## Background

The genomes of all organisms have been subjected to the deleterious effects of various environmental and metabolic factors since their origin. The integrity of the genomes has always been challenged by the influences of these agents, and therefore evolution provided organisms with several DNA repair pathways, which not only ensure the protection of cells against the lesions, but also guarantee the transmission of genetic information through the generations [1]. It has been reported that DNA damage results in various perturbations of physiological processes, such as reduced transcription and protein synthesis, destruction of phospholipid membranes and abnormalities in the cell cycle, which ultimately affect the development and growth of the organism [2]. It is also well known that the biological impact of any DNA mutagenic agent artificially used for the creation of genetic diversity depends on the chemical nature of the induced lesions and on the efficiency and accuracy of their repair. Although much has been learned from microbes and mammals about both the repair of DNA damage and the biological effects of the persistence of the lesions, much remains to be learned about the mechanism of DNA repair in plants [3]. Most frequently, DNA repair mechanisms are divided into several categories, such as photoreactivation, base excision repair, nucleotide excision repair, mismatch repair, non-homologous end joining, homologous recombination and damage-tolerance pathways [4].

Studies conducted mainly using bioinformatics tools have enabled identification of groups of genes participating in different pathways of DNA damage repair in Arabidopsis. However, information regarding these mechanisms in crop plants is very limited, primarily because functional approaches are based mainly on complete genomic sequences, which in this case are often unavailable. The high conservation level of Arabidopsis sequences related to DNA repair with respect to homologous genes in other species makes these sequences suitable as the queries for browsing the databases. However, searching for the homologs of Arabidopsis genes involved in DNA replication and repair is particularly difficult in the species whose complete genomic sequences are unavailable. One of the solutions is the use of ESTs as the basis for gene identification. The ESTs are defined as the fragments of mRNA sequences obtained through single-sequencing reactions that are performed on randomly selected clones from cDNA libraries. To date, over 45 million ESTs have been generated from over 1400 different eukaryotic species. EST projects are primarily used to either complement the existing genome projects or to serve as alternatives for the purposes of gene discovery [5]. The technology of EST sequencing offers a relatively inexpensive alternative to whole genome sequencing and has become a valuable resource for the gene identification [6]. To date, several databases have been developed to provide useful tools for the rapid retrieval of ESTs derived from a range of species. A species-specific database containing ESTs derived from several tissues of plants growing under different conditions was developed for sugarcane as the result of the Sugarcane EST Project (SUCEST) [3]. The database was used for determining the putative sugarcane homologs of Arabidopsis genes that are known to be involved in DNA repair [3,4]. To date, no publicly available database related to DNA replication and repair in plants has been established. Therefore, the aim of our project was to develop an open-access tool that would provide information about the genes and proteins involved in DNA replication and repair in Arabidopsis as the model species, the spatial and temporal expression patterns of the genes, the functional domains of the proteins and protein interaction models. It was intended that this information would be used as input data to browse databases of monocot sequences in order to identify homologous genes. In the database sequence information is provided for two monocot model species (*O. sativa* and *B. distachyon*) as they are used a reference in functional genomics; and for barley, which belongs to the crop species of crucial importance for world’s agriculture and food production.

Present paper describes the development of a database (bEST-DRRD) containing the ESTs and genomic sequences derived from four large barley source databases: HarvEST, TIGR, The IPK Crop EST (CR-EST) and the Computational Biology and Functional Genomics Laboratory. The database additionally encompasses two fully sequenced genomes of *O. sativa* and *B. distachyon*. The database content may be browsed using the Arabidopsis nucleotide and amino-acid sequences, which are involved in DNA replication and repair, as the queries. The sequence records of Arabidopsis genes were downloaded from the NCBI (National Center for Biotechnology Information) GenBank database (http://www.ncbi.nlm.nih.gov) [7]. The Arabidopsis genes regulating DNA replication are grouped into ten categories representing the consecutive steps of DNA replication [8]. Eight categories were assigned in order to reflect the diverse DNA repair processes (Table 1). To the best of our knowledge, this database is the first repository dedicated to the processes of DNA replication and repair in plants and is currently being applied as a tool for the identification of the genes involved in the above-mentioned mechanisms.

**Table 1:** The list of categories to which Arabidopsis sequences used as the queries were ascribed

**Process**	**Pathway or Protein Complex**	**No. of Arabidopsis genes**	**No. of mRNA variants**
**DNA replication**	Initiation	6	6
	Elongation	11	14
	Maturation	5	7
	ssDNA binding	9	12
	Helicase loading factors	4	5
	Replicative helicases	6	6
	Origin recognition	8	9
	PCNA loading complex	5	6
	GINS complex	5	7
	POLD clamp	2	2
**DNA repair**	Damage response	8	15
	Photoreactivation	6	9
	Rad6 pathway	9	17
	Non-Homologous End Joining	4	6
	Mismatch repair	11	11
	Base Excision Repair	17	24
	BER-related genes	5	9
	Nucleotide Excision Repair	27	37
**Total**		**148**	**202**

## Construction and content

**Figure 1:**
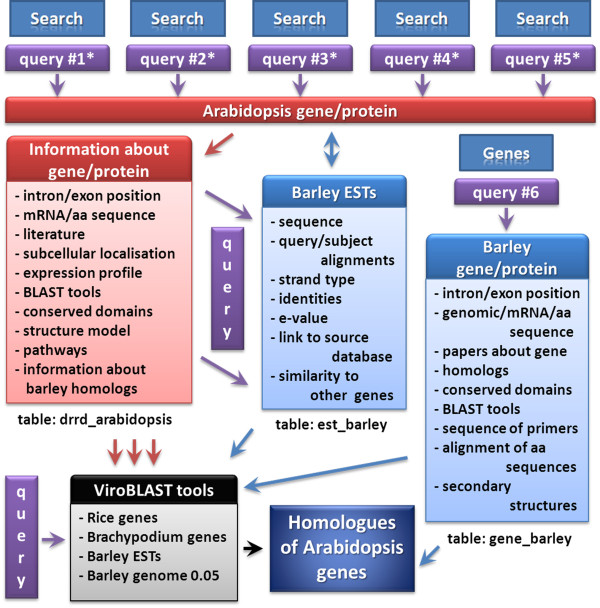
**The structure of the bEST-DRRD with the content of each component**. Arrows denote the direction from input sequence/information to the outputs. Asterisks indicate that query sequence may be selected on various levels of the database browsing, because Arabidopsis sequences, which are used as the queries, were categorized and grouped based on the DNA replication or repair process they mediate. Details are given in the text.

The phpMyAdmin program and the MySQL system were used for the construction of the bEST-DRRD. The database consists of three individual tables: the first one describes the Arabidopsis genes involved in DNA repair and replication (table name: drrd_arabidopsis), the second one describes barley ESTs similar to Arabidopsis genes (table name: est_barley) and the third one shows the barley genes cloned in the course of the project implementation (table name: gene_barley). Each table was designed specifically for each one of these three groups (Figure 1). The table for Arabidopsis genes contains information about the gene function, number of mRNA molecules produced during alternative splicing, all mRNA and coding sequences, the amino-acid sequences and the length of the proteins, as well as the accession numbers of all these entries in the NCBI database. The table for ESTs contains the data about the source of each EST, the sequence of EST, the alignment of strands, the identities (similarity shared with the query), the Expect value, as well as the start and stop positions of the alignment between the Arabidopsis sequence (query) and the barley EST (subject). In the table designed for the barley genes identified and cloned in the project, information about genomic, coding and amino acid sequences are provided, together with their NCBI GenBank accession numbers, the ESTs used for the gene identification and the primers that were used during the cloning of the gene. All the tables were linked by dedicated key entries that enable the identification of a single row in each table and connect it with similar rows in the whole bEST-DRRD. This resolution ensures the elastic scanning of the database content and simultaneous browsing of the content of different tables (Figure 1). Designing an individual table for each data collection enabled fast and easy modification of the database structure and the addition of new columns into the table.

The first step of the data gathering was searching the NCBI GenBank database for the Arabidopsis sequences and encoded polypeptides that are known to be involved in DNA replication and repair. The list of genes was assigned based on bioinformatic research and the analysis of literature data. To date, more than 200 Arabidopsis mRNA entries, including alternatively spliced versions of the transcripts, along with the sequences of encoded polypeptides, have been retrieved from the GenBank database. These sequences are used as the queries for browsing the repositories. Arabidopsis sequences along with encoded polypeptides were collected and categorised in a casual database. DNA replication-related sequences were arranged into ten groups based on the stage of the replication process they regulate: Origin recognition, Replicative helicases, Helicases’ loading factors, Initiation, GINS complex (a novel replication complex, the letters in the acronym stand for Go, Ichi, Nii, and San; five, one, two, and three in Japanese), Elongation, POLD (POLymerase Delta) clamp, PCNA (Proliferating Cell Nuclear Antigen) loading complex, Binding of ssDNA and Maturation. DNA repair and damage tolerance-related sequences were clustered according to the process they participate in: BER (Base Excision Repair), BER-related genes, NER (Nucleotide Excision Repair), MMR (Mismatch Repair), NHEJ (Non-Homologous End Joining), Photoreactivation, Rad6 pathway and damage response, which may be defined as the mechanism of DNA damage recognition, and propagating the signal to arrest the cell cycle and allow DNA repair (Table 1). The second part of bEST-DRRD structure is based on the ViroBLAST platform, which was developed as a sequence alignment web server by Prof. James Mullins and his co-workers at the University of Washington, Seattle, USA [9] and the NCBI C++ toolkit (ver. 2.2.25+). This tool was equipped with an access to several data sources in our database: Barley Genome version 0.05, containing 1 470 315 sequences that covers ca. 90% of the barley genome coding sequence, which was downloaded from http://harvest-web.org/utilmenu.wc, and Barley ESTs Assembly 35 that contains 50 937 sequences, which was obtained from http://harvest-web.org. Access to both repositories was kindly granted by Prof. Timothy J. Close from University of California, Riverside, USA. The rest of the repository encompasses the contents of open-access databases of the complete genomic sequences of *O. sativa*, derived from RGAP 7 – the Rice Genome Annotation Project (http://www.rice.tigr.org), and *B. distachyon* (http://www.brachypodium.org). The repository section dedicated to the rice genome includes more than 55 000 sequences. In the presented database, two components of the *B. distachyon* data source (downstream 1000 bp + upstream 1000 bp) were combined, resulting in more than 62 000 sequences. The ViroBLAST implements the NCBI C++ toolkit and may be used for a ‘basic search’ or an ‘advanced search’ method in which the search parameters may be customised. In order to establish the bEST-DRRD repository, the Arabidopsis sequences, which had been retrieved from the NCBI GenBank, were used as the queries to browse four barley sequence databases: HarvEST, TIGR Plant Transcript Assemblies (http://plantta.jcvi.org) [10], The IPK Crop EST Database (CR-EST) (http://pgrc.ipk-gatersleben.de/cr-est) [11] and the database of Computational Biology and Functional Genomics Laboratory (Gene Index Project) (http://compbio.dfci.harvard.edu) [12]. During ESTs retrieval from the TIGR and Gene Index Project databases, the BLASTN algorithm was applied with the matrix – blosum62, Expect value – 10 and alignments equal to 20. All the retrieved barley ESTs were annotated, categorised, grouped and ascribed to the query sequence.

## Utility and Discussion

### The bEST-DRRD interface

The bEST-DRRD website (http://www.best.us.edu.pl) home page interface contains several links. By choosing ‘Project’, the user can find general information about the project '*Mutational Analysis of Genes Involved in DNA Repair in Barley*', which is implemented in the Department of Genetics, University of Silesia and coordinated by the International Atomic Energy Agency (IAEA) in Vienna, Austria. The above-mentioned project is aimed at the identification of barley sequences homologous to the characterised Arabidopsis genes involved in the mechanisms of DNA repair as well as at the analysis of the identified sequences with the TILLING (Targeting Induced Local Lesions IN Genomes) strategy in order to isolate mutants that carry defects in this process. The link ‘Genes’ provides the list of barley genes that have been identified, cloned and characterised during the implementation of the project. These genes were published in NCBI GenBank database and are now being analysed functionally with TILLING. The link ‘BLAST’ directs the user to the ViroBLAST tool, where various databases of barley genomic and ESTs (derived from HarvEST project), rice and Brachypodioum genomic databases may be browsed using query sequences. It should be emphasised that in this step any input sequence (not necessarily related to DNA metabolism) may serve as a query. The HarvEST data source has been incorporated in the presented database because it is the main resource for barley ESTs and assemblies and many resources (Affymetrix GeneChip, Illumina Golden Gate Assay) are based on it. It contains highly curated ESTs (all based on raw data - not only FASTA). The barley ESTs derived from HarvEST are combined into unigenes and BLASTed relative to rice, Arabidopsis and UniProt. The link ‘Search’ enables the database to be browsed in order to find all Arabidopsis genes that are to be used as the queries. The user will find a short instruction on how the database may be screened and which categories are available (Table 1). The interface also provides links to the website addresses related to the project and the bEST-DRRD itself (‘Links’) and allows for feedback with the authors of the database (‘Contact’). The link ‘Team’ introduces the individuals involved in various tasks of the project, which are also listed.

### Browsing the database

The bEST-DRRD may be browsed using several different options (Figure 1). All the Arabidopsis genes from bEST-DRRD may be shown in the table in alphabetical order or the Arabidopsis genes, that are involved in DNA replication and DNA damage repair, may be displayed separately (also in a table and in alphabetical order). For each process (DNA replication and repair), the genes involved in distinct pathways, like Origin recognition or Base Excision Repair, may be displayed separately. The repository of Arabidopsis genes involved in DNA replication and repair may also be browsed using gene and/or protein names as well as the accession numbers from TAIR (The Arabidopsis Information Resource). For each Arabidopsis gene listed in table the name, short name, function of the gene and the NCBI GenBank accession numbers of the transcript and encoded protein are provided. The NCBI GenBank accession numbers are directly linked to the corresponding entries in the NCBI database. The option ‘search’ in the penultimate column of the table allows the retrieval of the barley EST sharing a similarity with the query and derived from TIGR, CR-EST and Gene Index Project resources. The results are depicted in the form of bars aligned with the query. For each of these, the database provides detailed positions of the query-EST alignment (the numbers above the ESTs, which are depicted as blue bars, refer to the nucleotide positions within the EST), the total length of EST (in parenthesis), the Expect value of the alignment together with identities (percentage of similarity) between the query and EST. For each EST the accession number is provided, which is directly linked with the sequence of EST in the FASTA format, orientation of the strands in the query-EST alignment and a view of the alignment with the positions of the nucleotides in the query and the subject (EST). In the last column, ‘details’ about each gene and encoded protein are provided. For each gene, apart from its name and function, the gene structure, sequences of mRNA, CDS and encoded protein are provided (in the FASTA format) together with the total length of the protein sequence and their NCBI GenBank and TAIR accession numbers. The ‘Toolbox’ options are also available for each gene. The ‘Publications’ link provides a comprehensive list of papers on the given gene from the PubMed and Google Scholar. The sub-cellular localisation of the gene product as well as the spatial and temporal expression profile of each gene are provided through the Arabidopsis eFP Browser (from http://bar.utoronto.ca). Two additional BLAST tools allow the mRNA and/or protein sequence to be used as the queries to search against NCBI GenBank database (BLASTN and BLASTP, respectively) for potentially homologous sequences from other species. The ‘Toolbox’ also provides models of conserved domains for proteins, derived from the Conserved Domains source of the NCBI database, and the putative secondary-structure models of the proteins from ModBase: the Database of Comparative Protein Structure Models (http://modbase.compbio.ucsf.edu/modbase-cgi/index.cgi). The ‘Toolbox’ also contains a description of the pathway that is mediated by the protein of interest. The data is derived from the BioSystems repository of the NCBI database (http://www.ncbi.nlm.nih.gov/biosystems).

The bEST-DRRD as a source of information on sequences related to DNA replication and repair in plants

The presented database contains the barley coding sequences that were identified using the database as a tool. The sequences of these barley genes had been confirmed after gene cloning. For each of the above genes additional information and options have been provided, that allow among others for a rapid search for the most conserved Eukaryotic homologs using the ‘HomoloGene’ tool of the NCBI database. Additionally, the ‘Toolbox’ provides a model of the conserved domains, for each barley protein, derived from the Conserved Domains source of the NCBI GenBank database. Similar to the Arabidopsis ‘Toolbox’, two additional BLAST tools allow the mRNA and/or protein sequence to be used as queries to search against the NCBI GenBank database (BLASTN and BLASTP, respectively) for any potentially homologous sequences. Moreover, the sequences of barley ESTs which were used as a basis for the gene cloning are available, together with the PCR primers applied during the procedure. The alignments of homologous protein sequences from barley, rice and Arabidopsis are provided, where conserved functional domains are depicted in colors with their respective domain codes. The database also includes models of secondary structure predictions performed using the PSIPRED Protein Structure Prediction Server [13] for barley, rice and Arabidopsis protein homologs.

The database is not intended merely as a repository of barley ESTs and therefore it may serve as a source of information on the genes, proteins and mechanisms of DNA-related processes in Arabidopsis as well. The presented database is based on query sequences derived from Arabidopsis, because in this species the mechanisms underlying DNA replication and repair have been described to the greatest degree. Only a few genes involved in the DNA repair process have been characterised and their functions have been functionally validated in monocot crops, including rice [14]. Therefore, the Arabidopsis sequences involved in DNA repair that have been identified so far can serve as the basis for the retrieval of sequences collected in other species databases in order to identify homologous genes. Moreover, the contents of the open-access databases (i.e. eFP Browser), which provide information about gene expression profiles (including DNA replication and repair-related genes), are by far more extensive for Arabidopsis than for any other plant species. This makes Arabidopsis the most suitable model for the computational characterisation of any group of genes, especially because DNA replication and repair mechanisms are highly conserved across many evolutionarily divergent phylogenetic groups. The data concerning the functional characterisation and expression profiles of Arabidopsis genes may therefore serve as cues for identifying the same features in other plant species.

Mutagenic techniques are very efficient tools that are required to develop necessary germplasm collections in model and crop species that facilitate the discovery of desired loci and alleles. Various mutation techniques are applied for the analysis of gene function. One of the powerful strategies of functional genomics is TILLING approach, which is currently applied for analysis of the cloned barley genes. TILLING generates an allelic series of mutations and provides a range of phenotypic severity, therefore it is often preferable in basic research because it allows a more informative insight into the function of the gene and its product than insertional mutagenesis [15,16]. Induction of mutations within the genes involved in DNA repair may alter the efficiency of this process and shed light on the molecular mechanism of DNA repair in plants. The bEST-DRRD is the first database, which is designed to provide data on functional characterisation of genes related to DNA replication and repair in monocot crop species.

## Conclusions

The presented barley EST DNA Replication and Repair Database was designed and established to provide an open-access tool for the identification of monocot homologs of the Arabidopsis genes that are responsible for DNA replication and repair. It is intended to constitute a helpful tool for browsing the barley, rice and *B.distachyon* sequence repository using sequences related to DNA replication and repair. This is the first database dedicated to these processes that collects data from several sources of EST and genomic sequences. The presented database offers a suitable tool that enables a more efficient identification of the genes related to DNA repair and will be a source of information on new alleles of the identified genes for the scientific community.

## Availability and requirements

The presented barley EST DNA Replication and Repair Database (bEST-DRRD) is available at the web address: http://www.best.us.edu.pl. This is a completely open-access database that was established at the server of the University of Silesia, Katowice, Poland. There are no restrictions to its use by non-academics.

## Links

The University of Silesia [http://www.us.edu.pl]

HarvEST [http://www.harvest.ucr.edu]

TIGR Plant Transcript Assemblies [http://plantta.jcvi.org]

The Computational Biology and Functional Genomics Laboratory (Gene Index Project) [http://compbio.dfci.harvard.edu]

The IPK Crop EST Database (CR-EST) [http://pgrc.ipk-gatersleben.de/cr-est]

Rice Genome Annotation Project [http://www.rice.tigr.org]

Brachypodium.org [http://www.brachypodium.org]

ViroBLAST [http://www.indra.mullins.microbiol.washington.edu]

## Competing interests

The authors declare that they have no competing interests

## Authors’ contributions

DG contributed to the conception and design of the database and the website, collected and categorised the sequence data, participated in the retrieval and categorisation of ESTs and drafted the manuscript. MM contributed to the design of the database, the website and the Internet platform, participated in the retrieval and categorisation of ESTs, established and updated the database and participated in drafting the manuscript. IS contributed to the conception of the database and participated in revising the manuscript. All authors read and approved the final manuscript.

## References

[B1] Singh SK Roy S Choudhury SR Sengupta DN DNA repair and recombination in higher plants: insights from comparative genomics of arabidopsis and rice BMC Genomics 2010 11 443 10.1186/1471-2164-11-443 20646326 PMC3091640

[B2] Britt AB Molecular genetics of DNA repair in higher plants Trends Plant Sci 1999 4 20 25 10.1016/S1360-1385(98)01355-7 10234266

[B3] Lima WC Medina-Silva R Galhardo RS Menck CFM Distribution of DNA repair-related ESTs in sugarcane Genet Mol Biol 2001 24 141 146

[B4] Costa RMA Lima WC Vogel CIG Berra CM Luche DD Medina-Silva R Galhardo RS Menck CFM Oliveira VR DNA repair-related genes in sugarcane Expressed sequence tags (ESTs) Genet Mol Biol 2001 24 131 140 10.1590/S1415-47572001000100018

[B5] Parkinson J Blaxter M Expressed sequence tags: an overview Methods Mol Biol 2009 533 1 12 10.1007/978-1-60327-136-3_1 19277571

[B6] Lindlöf A Gene identification through large-scale EST sequence processing Appl Bioinformatics 2003 2 123 129 15130797

[B7] Maglott D Ostell J Pruitt KD Tatusova T Entrez Gene: gene-centered information at NCBI Nucleic Acids Res 2007 35 D26 31 10.1093/nar/gkl993 17148475 PMC1761442

[B8] Shultz RW Tatineni VM Hanley-Bowdoin L Thompson WF Genome-wide analysis of the core DNA replication machinery in the higher plants Arabidopsis and rice Plant Physiol 2007 144 1697 1714 10.1104/pp.107.101105 17556508 PMC1949880

[B9] Deng W Nickle DC Learn GH Maust B Mullins JI ViroBLAST: A standalone BLAST web server for flexible queries of multiple databases and user's datasets Bioinformatics 2007 23 2334 2336 10.1093/bioinformatics/btm331 17586542

[B10] Childs KL Hamilton JP Zhu W Ly E Cheung F Wu H Rabinowicz PD Town CD Buell CR Chan AP The TIGR Plant Transcript Assemblies database Nucleic Acids Res 2007 35 D846 851 10.1093/nar/gkl785 17088284 PMC1669722

[B11] Künne C Lange M Funke T Miehe H Thiel T Grosse I Scholz U CR-EST: a resource for crop ESTs Nucleic Acids Res 2005 33 D619 621 15608274 10.1093/nar/gki119 PMC540073

[B12] Pertea G Huang X Liang F Antonescu V Sultana R Karamycheva S Lee Y White J Cheung F Parvizi B Tsai J Quackenbush J TIGR Gene Indices clustering tools (TGICL): a software system for fast clustering of large EST datasets Bioinformatics 2003 19 651 652 10.1093/bioinformatics/btg034 12651724

[B13] Bryson K McGuffin LJ Marsden RL Ward JJ Sodhi JS Jones DT Protein structure prediction servers at University College London Nucleic Acids Res 2005 33 W36 38 10.1093/nar/gki410 15980489 PMC1160171

[B14] Kimura S Tahira Y Ishibashi T Mori Y Mori T Hashimoto J Sakaguchi K DNA repair in higher plants; photoreactivation is the major DNA repair pathway in non-proliferating cells while excision repair (nucleotide excision repair and base excision repair) is active in proliferating cells Nucl Acids Res 2004 32 2760 2767 10.1093/nar/gkh591 15150342 PMC419598

[B15] Till BJ Reynolds SH Greene EA Codomo CA Enns LC Johnson JE Burtner C Odden AR Young K Taylor NE Henikoff JG Comai L Henikoff S Large-scale discovery of induced point mutations with high-throughput TILLING Genome Res 2003 13 524 530 10.1101/gr.977903 12618384 PMC430291

[B16] Stemple DL TILLING - a high-throughput harvest for functional genomics Nature Rev Genet 2004 5 145 150 10.1038/nrg1273 14726927

